# Effects of childhood trauma on aggressive behaviors and hippocampal function: the modulation of COMT haplotypes

**DOI:** 10.1093/psyrad/kkad013

**Published:** 2023-09-06

**Authors:** Chao Wang, Linfei Zhu, Wenyu Zheng, Hanyuzhu Peng, Jiaojian Wang, Yue Cui, Bing Liu, Tianzi Jiang

**Affiliations:** School of Psychology, Shenzhen University, Shenzhen 518060, China; School of Psychology, Shenzhen University, Shenzhen 518060, China; School of Psychology, Shenzhen University, Shenzhen 518060, China; School of Psychology, Shenzhen University, Shenzhen 518060, China; State Key Laboratory of Primate Biomedical Research, Institute of Primate Translational Medicine, Kunming University of Science and Technology, Kunming 650500, China; Brainnetome Center, Chinese Academy of Sciences, Beijing 100190, China; National Laboratory of Pattern Recognition, Institute of Automation, Chinese Academy of Sciences, Beijing 100190, China; State Key Laboratory of Cognitive Neuroscience and Learning, Beijing Normal University, Beijing 100875, China; Brainnetome Center, Chinese Academy of Sciences, Beijing 100190, China; National Laboratory of Pattern Recognition, Institute of Automation, Chinese Academy of Sciences, Beijing 100190, China

**Keywords:** childhood trauma, COMT, hippocampus, aggression

## Abstract

**Background:**

Aggression is a commonly hostile behavior linked to the hippocampal activity. Childhood trauma (CT) exposure has been associated with altered sensitization of the hypothalamic-pituitary-adrenal (HPA) axis and hippocampal volume，which could increase violent aggressive behaviors. Additionally, Catechol-O-methyltransferase (COMT), the major dopamine metabolism enzyme, is implicated in stress responsivity, including aggression. Hence, CT exposure may affect aggression through the effect on the hippocampal function, which might also be modulated by the COMT variations.

**Objectives:**

This study examined whether both CT and haplotypes of COMT moderate hippocampal function and thus affect human aggressive behavior.

**Methods:**

We obtained bilateral hippocampal functional connectivity maps using resting state functional magnetic resonance imaging (MRI) data. COMT haplotype estimation was performed using Haploview 4.2 and PHASE 2.1. Then we constructed a moderated mediation model to study the effect of the CTQ × COMT on aggressive behavior.

**Results:**

Three major haplotypes were generated from thirteen single nucleotide polymorphisms (SNPs) within the COMT gene and formed three haplotypes corresponding to high, medium, and low enzymatic activity of COMT. The results showed interactive relationships between the Childhood Trauma Questionnaire (CTQ) and COMT with respect to the functional connectivity (FC) of the bilateral hippocampus (HIP)-orbital frontal cortex (OFC). Specifically, CT experience predicted lower negative HIP-OFC coupling in the APS and HPS haplotypes corresponding to the medium and high enzymatic activity of COMT, but greater FC in the LPS haplotypes corresponding to the low enzymatic activity. We also observed a conditional mediation effect of the right HIP-OFC coupling in the link between COMT and aggressive behavior that was moderated by CT experience.

**Conclusions:**

These results suggest that CT and COMT have a combined effect on aggressive behavior through hippocampal function. This mediation analysis sheds light on the influence of childhood experience on aggressive behavior in different genetic backgrounds.

## Introduction

Aggression is defined as any behavior toward another individual through physical actions or words, and so on, that is carried out with the intent to cause physical or psychological harm (Nelson & Trainor, 2007). The aggression will cause multiple undesirable problems, such as interpersonal tensions, increased suicides, and increased criminal activities (Lischinsky & Lin, 2020). The neural basis of aggression has been extensively investigated, which is an imbalance between the “top-down” control of the prefrontal cortex and excessive “bottom-up” activity of the limbic regions (Yang *et al*., 2010). Specifically, both human and animal studies have indicated the correlation between the hippocampus (HIP) and the regulation of aggressive behaviors (Chang & Gean, 2019; Sala *et al*., 2011). The HIP, together with the amygdala and prefrontal cortex, is implicated in the processing of social emotions, fear, and aggressive reaction (Cupaioli *et al*., 2021), the reduction in the gray matter volume of the HIP and amygdala were associated with elevated aggression severity (Coccaro *et al*., 2015). Previous studies also found that tumors or infections in the HIP could be considered as triggering causes for aggressive behaviors (Malamud, 1967). The higher activation in the HIP was also related to the overreactions to threatening stimuli (Lee *et al*., 2009). In addition, human aggressive behaviors are promoted by multiple environmental events, especially childhood trauma (CT). Studies have found that childhood abuse experience is related to violent aggressive behavior, more precisely, psychological disorders, aggressive behavior, and behavioral problems of childhood traumatized children are more than those of non-traumatized children (Dodge *et al*., 2003).

Childhood trauma refers to an adverse experience, including psychological, physical, or sexual abuse, and various forms of neglect during childhood (Bernstein *et al*., 2003). CT exposure contributes to the exhibit of aggressive behavior in adolescence, which also predicts aggressive and anti-social behaviors of adults (Cupaioli *et al*., 2021). CT has also been associated with sensitization of the hypothalamic–pituitary–adrenal (HPA) axis (Heim *et al*., 2008) and volumetric abnormalities in the HIP (Janiri *et al*., 2017). The HPA axis is the major neuroendocrine stress response system, and hippocampal neuroplasticity is critically linked to the stress-related regulation of HPA axis activity (Snyder *et al*., 2011). Evidence from animal and human studies suggested that hippocampal growth mainly occurs during childhood and that early stress exposure has lasting effects on the normal functioning of the HIP–HPA axis (Tottenham & Sheridan, 2009). Greater hippocampal activation has been linked to enhanced sensitivity while processing threatening information in people with a history of CT (Edmiston & Blackford, 2013).

Moreover, aggressiveness is also linked to the dysregulation of the dopamine (DA) system. The DA system is involved in rewarding processing and goal-directed behavior, Overreaction and greater aggression in the response to provocation are linked to the decreased DA synthesis. Catechol-*O*-methyltransferase (COMT) is a major enzyme responsible for degrading DA. The COMT gene, mapped to 22q11.1-q11.2, is widely expressed in the prefrontal cortex and other regions that modulate aggressive behavior (Matsumoto *et al*., 2003). The most investigated functional single nucleotide polymorphism (SNP) of the COMT gene, which is in codon 158, codes for a substitution from valine (Bernstein *et al*., 2003) to methionine (Boecker-Schlier *et al*., 2016), leading to a three- to four-times reduction in the activity of the COMT enzyme (Bilder *et al*., 2004). The low COMT activity leads to elevated dopamine level and thus leads to increased stimulation of dopamine neural networks implicated in impulsivity and aggressiveness such as prefrontal cortex and limbic areas (Cupaioli *et al*., 2021; Drabant *et al*., 2006). There was a higher risk for aggressive behavior in healthy young people and schizophrenia patients carrying the Met allele relative to the Val homozygotes (Albaugh *et al*., 2010; Bhakta *et al*., 2012). In addition, previous studies also have concluded that COMT Val158Met is associated with hippocampal activity (Krach *et al*., 2010; Wang *et al*., 2018) and its functional connectivity (FC) with the prefrontal cortex during human cognitive performance (Laatikainen *et al*., 2012; Meyer *et al*., 2016). During emotion processing and memory tasks, the Met^158^ was associated with greater connectivity between HIP and prefrontal regions (Klucken *et al*., 2015; Morris *et al*., 2020). The hippocampal reactivity to unpleasant stimuli is also correlated with the number of Met^158^, which might contribute to differences in emotional resilience under adverse conditions (Smolka *et al*., 2005). Considering the effect of COMT on the aggression and hippocampal function, it is reasonable to infer that COMT might modulate the effect of environment adversity on the hippocampal function and aggressive behaviors.

The interaction between the environment and COMT Val158Met has been found to influence hippocampal volume in healthy adults (Rabl *et al*., 2014) and hippocampal activation in resilient individuals (van Rooij *et al*., 2016), but these interaction effects have not always been detected (Batalla *et al*., 2018). Different measurements of environment factors between samples may be a possible reason for this inconsistency. According to a previous study, compared with single SNP analysis, haplotype analysis improved the statistical power for the moderating effect of COMT haplotypes on the association between antenatal maternal anxiety and neonatal cortical morphology (Qiu *et al*., 2015). Hence, the modest effect of an individual SNP combined with neglecting associated SNPs might be another potential reason. However, COMT Val158Met cannot be the only explanation for the variability in the activity of the COMT enzyme. This SNP, together with SNPs rs6269, rs4633, and rs4818, can be integrated into three major COMT haplotypes (HPS, APS, and LPS) corresponding to high, medium, and low COMT activity (Diatchenko *et al*., 2006). Although based on different molecular genetic mechanisms, the APS and HPS haplotypes, respectively, lead to a three times and a roughly 20 times reduction in COMT enzymatic activity compared with the LPS haplotype (Nackley *et al*., 2006). Hence, COMT haplotypes other than COMT Val158Met seem to have a very powerful effect on brain function and behavior.

The interaction between adverse experience during childhood and COMT Val158Met has been found to affect adult antisocial behavior, such as aggressive and violent behavior (Hygen *et al*., 2015). But, to our knowledge, although altered hippocampal volume and activity have been shown to correlate with impulsive aggression (Visser *et al*., 2014), little evidence has been presented about which neural substrate is involved in the way that this gene–environment interaction is related to aggressive behavior.

Because the potential gene–environment interaction predicts aggressive behavior and because both CT and aggression have been associated with abnormal hippocampal activity, we speculated that the interaction between CT and COMT haplotypes would affect hippocampal function and that this region would mediate the gene–environment interaction on aggressive behavior. We first identified the three major COMT haplotypes and then investigated the resting-state FC of the bilateral HIP to reveal the neural substrates underlying this mediation effect.

## Materials and Methods

### Participants

A total of 360 healthy Han Chinese participants (186 males and 174 females, mean age 19.41 ± 1.09 years) were recruited. This study was approved by the Ethics Committee of the School of Life Science and Technology, University of Electronic Science and Technology of China. Written informed consent was obtained from each participant, and each was screened using the structured clinical interview for DSM-IV-TR Axis I disorders, Chinese edition (Phillips & Liu, 2011) to ensure that they had no current or lifetime diagnosis of Axis I mental illnesses or family history of psychiatric disorders. The exclusion criteria also included contraindications to magnetic resonance imaging (MRI) screening, traumatic brain injury, or brain lesions.

### DNA extraction, SNP genotyping, and haplotype analysis

We extracted genomic DNA from whole blood using the E.Z.N.A.™ Blood DNA Kit (Omega Bio-Tek, GA). Thirteen known SNPs within the COMT gene (rs2075507, rs737865, rs174675, rs174690, rs740603, rs4646312, rs6269, rs4633, rs4818, rs4680 (Val158Met), rs4646316, rs174699, and rs165599) were genotyped via the iPLEX Gold method (Sequenom, San Diego, CA, USA), using primers designed with Sequenom Assay Design v.3.1 software. Each sample was genotyped in duplicate to ensure accuracy. Alleles were automatically called with Sequenom's MassARRAY Typer v.4.0 software and checked by two independent workers. All COMT SNPs passed the criteria of a call rate of >90%, minor allele frequency of >0.05, and Hardy–Weinberg equilibrium of *P* > 0.05.

COMT haplotype estimation was performed using Haploview v.4.2 and PHASE v.2.1 (Stephens & Scheet, 2005). Using the linkage disequilibrium (LD) relationship between SNPs, we calculated the major haplotypes and their frequency in our participant group using Haploview software. PHASE v.2.1 was further used to estimate the most likely haplotype combination for each participant with a degree of confidence >90%.

**Figure 1:**
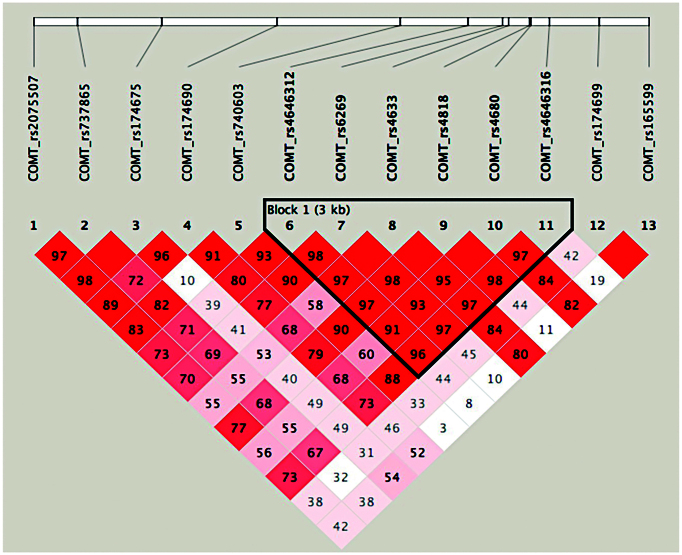
LD map of the 13 SNPs in our sample. Using varying shades of red and the *D*' statistic value, the map shows the LD level of the corresponding two SNPs.

The LD analysis showed that the significant haplotype block comprised six SNPs (rs4646312, rs6269, rs4633, rs4818, rs4680, rs4646316) in the strong LD (mean pairwise *r*^2^ = 0.976, see Fig. 1). Two major haplotypes, TACCGC and TATCAC, respectively, corresponded to the HPS and APS haplotypes (Nackley *et al*., 2006). The frequencies were 34.8% for HPS and 28.0% for APS. Another two haplotypes (31.9% for CGCGGT and 1.30% for CGCGGC) corresponded to the LPS and the above four haplotypes accounted for 96% of all the detected haplotypes. Each pair of haplotypes from the pair of homologous chromosomes was combined to form a diplotype that could provide more complete genetic information. These three major haplotypes formed six diplotypes (LPS/LPS, LPS/APS, LPS/HPS, APS/APS, APS/HPS, and HPS/HPS). We excluded 25 participants who carried none of the six diplotypes from the subsequent imaging genetic analyses. Due to the relatively small sample size of the haplotype homozygotes of LPS, APS, and HPS, we combined them with LPS/APS, LPS/HPS, and APS/HPS, respectively, and defined them as LPS haplotypes (52 males and 44 females), APS haplotypes (54 males and 57 females), and HPS haplotypes (49 males and 54 females), corresponding to the high, medium, and low enzymatic activity of COMT (Diatchenko *et al*., 2006; Nackley *et al*., 2006).

### Childhood trauma and aggression assessments

Childhood trauma was assessed with the Childhood Trauma Questionnaire (CTQ) (Bernstein *et al*., 1994), which is a self-report instrument that assesses CT in five areas: emotional, physical, and sexual abuse, and emotional and physical neglect. The CT score was calculated by the sum of all of these categories. As in the previous study (Frodl *et al*., 2014), we used a broad definition of CT according to the established cut-off score for each type of CT and classified participants as having a history of CT if they had scores greater than the cut-off value in at least one type of CT. Cut-off scores are as follows: ≥10 for physical abuse, ≥13 for emotional abuse, ≥8 for sexual abuse, ≥15 for emotional neglect, and ≥10 for physical neglect. Aggressive attitudes and behaviors were assessed using the Aggression Questionnaire (Buss & Warren, 2000), which consists of 34 items assessing aggressive proneness on five subscales: physical aggression, verbal aggression, indirect aggression, anger, and hostility. Also, according Buss and Perry (1992), physical and verbal aggression subscales are highly correlated with each other, both of which represent the individual engagement in aggressive behaviors; whereas anger and hostility subscales represent the tendency to have aggressive thoughts or feelings (Karlsgodt *et al*., 2015).

### MRI data acquisition

MRI scans were performed using an MR750 3.0 Tesla MR scanner (GE Healthcare, Milwaukee, WI, USA). Resting-state functional imaging data were acquired using a gradient-echo echo-planar imaging sequence with the following parameters: repetition time (TR) = 2000 ms, echo time (TE) = 30 ms, field of view = 240 × 240 mm^2^, matrix = 64 × 64, flip angle (Cupaioli *et al*., 2021) = 90°, voxel size = 3.75 × 3.75 × 4.00 mm^3^, 39 slices, and 255 volumes. Before the scanning, all the participants were asked to relax, think of nothing in particular, keep their eyes closed, and avoid sleeping. After scanning, the participants were asked whether they fell asleep to try to ensure that they kept awake during the entire scanning.

### fMRI analysis

All the raw functional MRI (fMRI) data were reviewed independently by two experienced radiologists who knew nothing about the genotype information. In total, 25 participants were excluded because of bad imaging quality, such as apparent signal loss and inter-slice motion artifacts. All the images were preprocessed using DPARSFA (Data Processing Assistant for Resting-State fMRI Advanced Edition, http://www.restfmri.net/forum/DPARSF), with the procedures as follows: (i) discarding the first 10 volumes; (ii) slice timing correction; (iii) head motion correction; (iv) spatial normalization to the EPI template in MNI space; (v) resampling to 3 × 3 × 3 mm^3^; (vi) smoothing with a 6 mm Gaussian kernel; (vii) regressing out nuisance signals including six motion parameters, white matter, cerebrospinal fluid signals, and global signals; and (viii) temporal bandpass filtering (0.01–0.8 Hz). A further 25 participants were excluded due to a maximum displacement in any of the cardinal directions (*x, y, z*) of >2 mm or a maximum spin (*x, y, z*) of >2°. The remaining 310 participants were included in the subsequent analyses.

The bilateral hippocampi were extracted as regions of interest from the Harvard–Oxford subcortical atlas (Desikan *et al*., 2006). The regions of interest masks were resampled to 3 × 3 × 3 mm^3^. Then, the Pearson correlation coefficients were calculated between the average time series of the bilateral hippocampi and those of all the voxels throughout the whole brain. The correlations were then converted to *z* values following Fisher's *r*-to-*z* transformation to improve normality. From these procedures we obtained bilateral hippocampal FC maps for each participant.

### Statistical analysis

The normality of CTQ and aggression scores were checked by means of skewness and kurtosis and the Kolmogorov–Smirnov test. Two-way analyses of variance (ANOVA) were performed with CTQ and COMT haplotypes as two independent variables and with age and sex as covariates to examine the effect of the CTQ × COMT haplotypes interaction on hippocampal function. These statistical steps were completed using Statistical Parametric Mapping 12 (http://www.fil.ion.ucl.ac.uk/spm). Multiple comparison corrections were performed using the Gaussian random field (GRF) method implemented in the Data Processing & Analysis for Brain Imaging (http://rfmri.org/DPABI). The significance level was defined as those clusters that survived a threshold of *P* < 0.01 (cluster-forming threshold at voxel-level *P* < 0.001, and cluster level *P* < 0.01). The significant region, as identified by the two-way ANOVAs based on hippocampal FC, was extracted and the *z* values of the voxels in the region were averaged for each participant. Two-way ANOVAs and simple effect analyses were performed using SPSS v.20.0. Then, to determine the relationship between FC and aggression, Pearson correlation coefficients were measured between the mean *z* values and the aggressive scores. Next, a moderated mediation model was constructed to test whether the effect of the CTQ × COMT haplotypes interaction on aggressive behavior could be mediated by the hippocampal whole brain connectivity. We used the SPSS version of the PROCESS macro (www.afhayes.com) (Hayes, 2012) to test the mediation model (model 8), with the CTQ results as the predictor, COMT haplotypes as the moderator, aggression score as the outcome variables, the average hippocampal FC as the mediator, and age and sex as covariates. The significance level was established by 95% confidence intervals (Ansell *et al*., 2012) with 50 000 bootstrapped iterations.

## Results

### Demographics and genetic characteristics

Although the Kolmogorov–Smirnov test showed that the aggression scores was violated the assumption of normality (*z* = 0.05, *P* = 0.04), skewness and kurtosis were within the acceptable range of −1 to +1 (skewness 0.61, kurtosis 0.46). The CTQ results was not normally distributed (skewness 1.50; kurtosis 3.77; Kolmogorov–Smirnov test: *z* = 0.11, *P* < 0.001), and we treated it as a dichotomous variable to distinguish between the presence and absence of significant CT experience. The details of the demographics and genetic characteristics are shown in Table 1. Two-way ANOVAs and chi-square tests did not show any significant main effects of CTQ and COMT, or any interactive effect of CTQ × COMT across all groups in age, gender, and aggression thoughts (*P* > 0.05), except for the main effect of the CTQ on aggressive behaviors (*P* < 0.05).

**Table 1:** Demographics: and genetic characteristics of the different groups.

CTQ	CT+			CT−		
COMT	LL&LA	LH&AA	AH&HH	LL&LA	LH&AA	AH&HH
Number	26	34	34	70	77	69
Male/female^a^	14/12	17/17	19/15	38/32	37/40	30/39
Age^b^ (SD)	19.47 (0.83)	19.62 (0.99)	19.48 (1.30)	19.38 (1.15)	19.45 (1.06)	19.27 (0.92)
Aggression total score^b^ (SD)	70.88 (16.92)	75.79 (18.82)	76.85 (14.81)	69.83 (16.90)	71.84 (17.14)	70.84 (15.57)
Aggressive behaviors measure^c^ (SD)	25.19 (7.77)	27.59 (8.23)	27.56 (8.02)	24.31 (7.38)	25.19 (6.62)	24.84 (6.61)
Aggressive thoughts measure^b^ (SD)	32.27 (7.30)	34.32 (9.08)	34.97 (7.50)	32.27 (8.45)	33.24 (8.70)	32.59 (8.20)

SD: standard deviation; LL: LPS/LPS; LA: LPS/APS; LH: LPS/HPS; AA: APS/APS; AH: APS/HPS; HH: HPS/HPS; CT+: the presence of significant CT experience; CT−: the absence of significant CT experience

a Chi-square test (*P* > 0.05) across the six groups;

b Two-way ANOVA (main effect of CTQ and COMT : *P* > 0.05; CTQ × COMT : *P* > 0.05);

c Two-way ANOVA (main effect of CTQ: *P* < 0.05; main effect of COMT: *P* > 0.05; CTQ × COMT : *P* > 0.05).

### Interactive effects of CTQ and COMT on hippocampal FC

**Figure 2:**
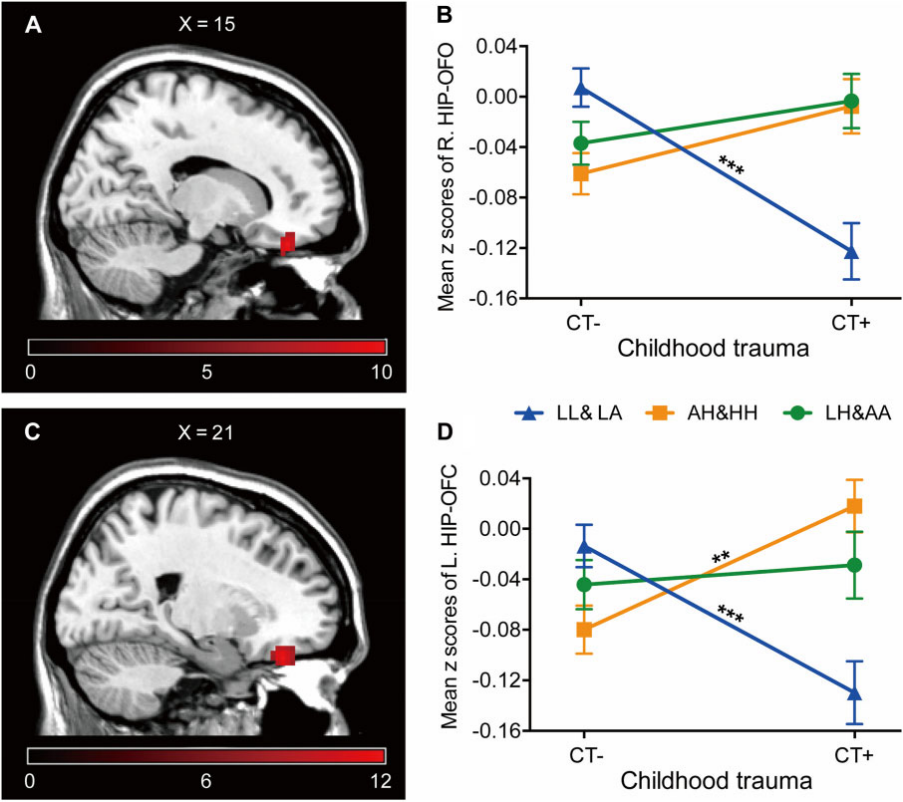
Interaction of COMT haplotypes and the CTQ results on bilateral hippocampal FC. (**A**) The significant region shows the effect of the CTQ × COMT interaction on the FC between the right HIP and right OFC (GRF correction, voxel level *P* < 0.001, cluster level *P* < 0.01). (B) Mean ± SE FC of R.HIP–OFC in (A) across all participants (*post hoc t*-test, Bonferroni corrected). (**C**) The significant region shows the effect of the CTQ × COMT interaction on the FC between the left HIP and right OFC (GRF correction, voxel level *P* < 0.001, cluster level *P* < 0.01). (D) Mean ± SE FC of R.HIP–OFC in (C) across all participants (*post hoc t*-test, Bonferroni corrected). R., right, L., left, SE, **P < 0.01, ***P < 0.001.

We found significant interactions of CTQ × COMT on the FC between the right HIP and the right orbital frontal cortex (OFC, MNI coordinates: 15, 39, −24; *Z*-score = 3.94, GRF correction, voxel level *P* < 0.001, cluster level *P* < 0.01, Fig. 2A). The FCs between the left HIP and right OFC were also affected by the CTQ × COMT interaction (GRF correction, voxel level *P* < 0.001, cluster level *P* < 0.01, Fig. 2C). No main effect for either the CTQ or the COMT results alone was identified by ANOVAs (GRF correction, voxel level *P* < 0.001, cluster level *P* < 0.01).

We further calculated mean value of *z* scores in the above regions that had significant intergroup differences. Specifically, the CTQ × COMT interaction had a significant influence on the hippocampal FCs (right HIP–OFC: *F* (2302) = 11.25, *P* < 0.001; left HIP–OFC: F (2302) = 10.33, *P* < 0.001). Simple effect tests indicated that, in individuals with high COMT enzymatic activity (LPS haplotypes), the presence of CT was associated with increased negative FC of the bilateral HIP–OFC compared with the absence of CT (right HIP–OFC: *t* = 4.55, *P* < 0.001, Fig. 2B; left HIP–OFC: *t* = 3.66, *P* = 0.001, Fig. 2D; Bonferroni corrected). In individuals with low COMT enzymatic activity, i.e. HPS haplotypes, the presence of CT was had the opposite effect (right HIP–OFC: *t* = 1.93, *P* = 0.062, Fig. 2B; left HIP–OFC: *t* = 3.18, *P* = 0.002, Fig. 2D; Bonferroni corrected).

### Moderated mediation analysis

**Figure 3:**
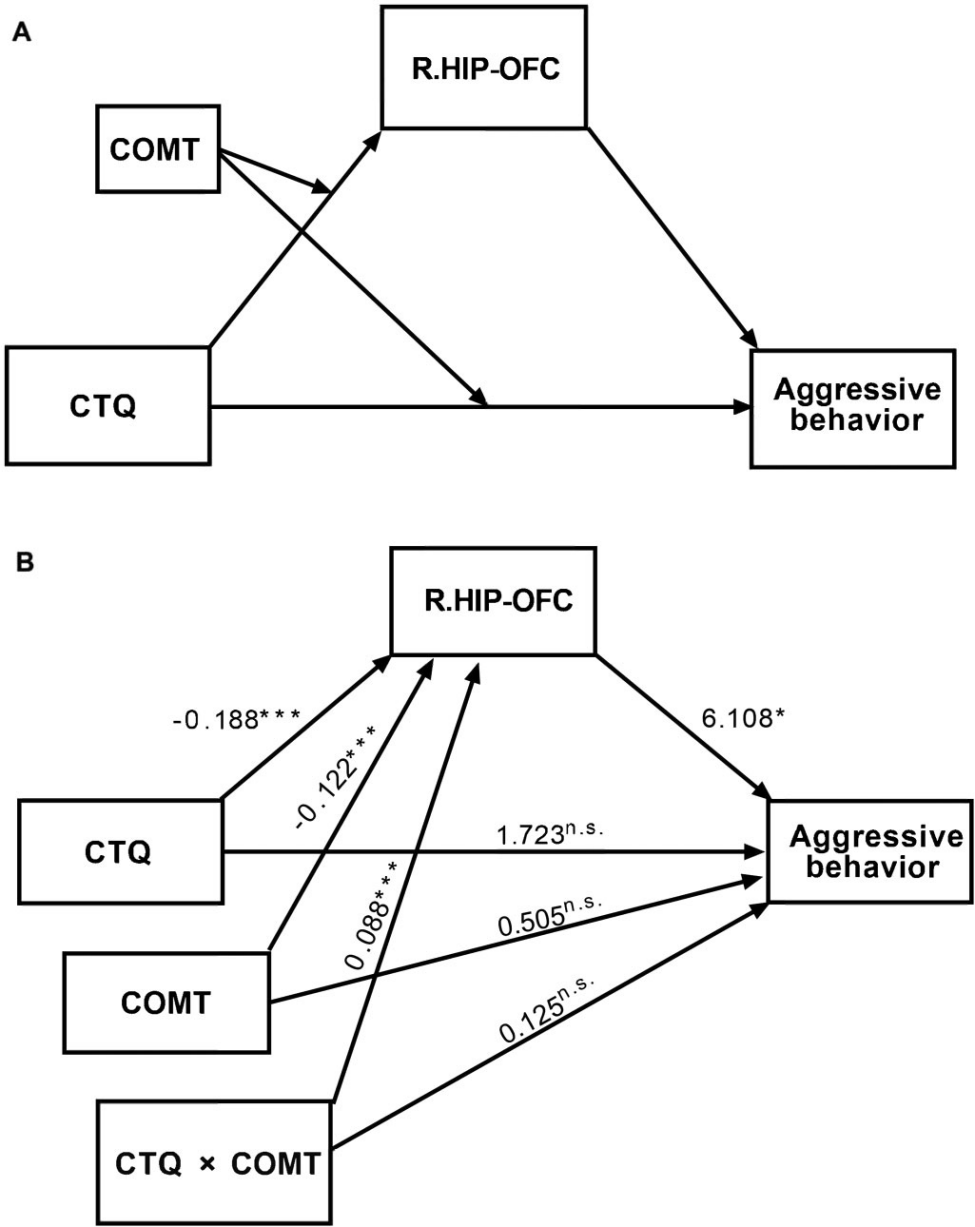
The moderated mediation analysis. (**A**) Moderated mediated model between CTQ, COMT haplotypes, FC of R.HIP–OFC, and aggressive behaviors. (**B**) Results of moderated mediated model between CTQ, COMT haplotypes, FC of R.HIP–OFC, and aggressive behaviors. The conditional direct effects of CTQ on aggressive behaviors was significant in individuals with APS haplotypes [LPS haplotypes: direct effect estimate = 1.848, SE = 1.482, 95% CI (−1.068, 4.765); APS haplotypes: direct effect estimate = 1.973, SE = 0.879, 95% CI (0.243, 3.702); HPS haplotypes: direct effect estimate = 2.098, SE = 1.375, 95% CI (−0.608, 4.803)]. The results showed a significant moderated mediation effect of the CTQ and COMT results on aggressive behaviors through the FC of the right HIP–OFC (indirect effect estimate = 0.538, SE = 0.285, 95% CI (0.035, 1.153)]. The conditional indirect effects of CTQ on aggressive behaviors through the FC of the right HIP–OFC were also significant in individuals with LPS haplotypes and with HPS haplotypes [LPS haplotypes: indirect effect estimate = −0.611, SE = 0.327, 95% CI (−1.317, −0.042); APS haplotypes: indirect effect estimate = −0.073, SE = 0.112, 95% CI (−0.317, 0.140); HPS haplotypes: indirect effect estimate = 0.465, SE = 0.283, 95% CI (0.021, 1.111)].

Before the mediation analysis, Pearson correlation coefficients were computed to examine the relationship between the FC and aggression. We only observed a significant and positive correlation between the *z* values of the right HIP–OFC and the aggressive behaviors measure (*r* = 0.122, *P* = 0.032). Considering the co-occurrent relationships between the CTQ × COMT interaction, hippocampal function, and the aggressive behaviors, a moderated mediation model was constructed to examine whether the FC of the right HIP–OFC mediated the effect of the CTQ × COMT interaction on the aggressive behaviors. The details of the moderated mediated model are presented in Table 2, and the overall model is illustrated in Fig. 3. The FC of the right HIP–OFC was significantly associated with the CTQ results (*β* = −0.188; *P* < 0.001) as well as with COMT (*β* = −0.122; *P* < 0.001) and with CTQ × COMT (*β* = 0.088; *P* < 0.001). Participants who had a high enzymatic activity of COMT and those reporting a history of CT experience were found to be more likely to have decreased FC. The aggressive behaviors measure was only significantly associated with the FC of the right HIP–OFC (*β* = 6.108; *P* = 0.043). Conditional direct effects of the CTQ on the aggressive behaviors (*β* = 0.505; *P* = 0.745) was only found in participant with APS haplotypes [direct effect estimate = 1.973, SE = 0.879, 95% CI (0.243, 3.702)].

**Table 2:** Results: of moderated mediation model.

R.HIP–OFC	*β*	*t*	*p*
CTQ	−0.188	−4.077	<0.001
COMT	−0.122	−4.223	<0.001
CTQ × COMT	0.088	4.211	<0.001
**Aggressive behaviors measure**			
R.HIP–OFC	6.108	2.032	0.043
CTQ	1.723	0.694	0.488
COMT	0.505	0.325	0.746
CTQ × COMT	0.125	0.111	0.912

Conditional indirect effect of *X* (CTQ) on *Y* (aggressive behaviors) through *M* (FC of R.HIP–OFC) = (−0.188 + 0.088W)*6.108;

Conditional direct effect of *X* (CTQ) on *Y* (aggressive behaviors) = 1.723 + 0.125*W*;

COMT haplotypes is the moderator represented by *W*.

Additionally, the results revealed a significant moderated mediation effect of the CTQ results and COMT on the aggressive behaviors through the FC of the right HIP–OFC [indirect effect estimate = 0.538, standard error (SE) = 0.285, 95% CI (0.080, 1.217)]. The conditional indirect effects of CTQ on the aggressive behaviors through the FC of the right HIP–OFC showed opposite directions in individuals with different levels of COMT enzymatic activity [LPS haplotypes: indirect effect estimate = −0.611, SE = 0.327 95% CI (−1.317, −0.042); APS haplotypes: indirect effect estimate = −0.073, SE = 0.112, 95% CI (−0.317, 0.140); HPS haplotypes: indirect effect estimate = 0.465, SE = 0.283, 95% CI (0.021, 1.111)].

## Discussion

This study revealed the associations between childhood trauma, COMT, hippocampal function, and aggressive behavior. The genetic effect was characterized by variations in the combination of the haplotypes composed of individual SNPs. We found that the CTQ results and the COMT haplotypes have interactive effects on the FC of the HIP–OFC. Our results were, to the best of our knowledge, the first to indicate that the regulation of childhood trauma experience on aggression through hippocampal FCs is moderated by COMT haplotypes. Such gene–environment interactions may partially explain how individual differences in environmental experience can modulate genetic influences on brain function and, consequently, on behavior.

Our study highlighted an interaction between the CTQ and COMT results on the FC between the bilateral HIP and the OFC. Consistent with a previous study (Cheng *et al*., 2017), the OFC was negatively connected to the bilateral hippocampi. The OFC is a key region involved in adaptation to environmental change and reward processing, as well as to emotional and social regulation (Boecker-Schlier *et al*., 2016). The HIP is involved in processing stress and regulating arousal and affective states. Animal studies suggested that stress can lead to alterations in dendritic arborization in the OFC (McEwen & Morrison, 2013), as well as attenuated long-term potentiation in the HIP (Alfarez *et al*., 2003). Human studies also found that adversity-related reductions in the volume and functional activity of these two regions may act as vulnerability markers that precede the presence of psychiatric symptoms, such as loss of self-control or fear extinction deficits (Ansell *et al*., 2012). These results indicate that the FC of the HIP–OFC might be affected by the environment, especially by adverse and stress conditions. A recent study reviewed the available researches that focused on the effect of COMT Val158Met on FC, they found that the Met^158^ was associated with an increased FC between HIP and OFC during resting state in healthy adults (Morris *et al*., 2020). To our knowledge, previous studies also have investigated the effect of COMT the influence of COMT Val158Met on PFC connectivity at rest and found a dose-dependent relationship between COMT Val^158^ allele and FC of PFC regions (Lee *et al*., 2011; Liu *et al*., 2010). Meanwhile, in light of the high level of expression of COMT in both the HIP and the frontal cortex, it is reasonable to find an interaction effect of the CTQ and COMT on the negative HIP–OFC couplings.

Specifically, the CT experience predicted lower negative HIP–OFC coupling in the APS and HPS haplotypes, whereas the CT experience predicted greater FC in the LPS haplotypes. Cell studies revealed that the LPS haplotype is associated with about three times higher enzymatic activity than the APS haplotype, which is most likely due to the substitution from the Val^158^ to the Met^158^ allele (Nackley *et al*., 2006). Hence, from this perspective, our findings were consistent with a previous study in which environmental adversity was associated with increased hippocampal volume in Val^158^ homozygotes, and this correlation exhibited a gradual change from positive to negative with the dose of the Met^158^ allele (Rabl *et al*., 2014). In addition, the moderating effect of COMT Val158Met on the relationship between antenatal maternal anxiety and neonatal cortical thickness showed a similar pattern (Qiu *et al*., 2015). COMT Val158Met also has a pleiotropic effect on cognitive and emotional processing, with the Val allele related to poor memory performance and with the Met allele related to decreased emotional regulation (Mier *et al*., 2010). Heightened connectivity in the LPS haplotype may indicate increased aversive memory formation and consolidation caused by CT experience (Mutso *et al*., 2014). Given the involvement of the OFC in the down-regulation of negative emotion, weakened connectivity in the APS haplotype with CT experience may indicate diminished control of negative emotions. Moreover, the imaging study found that the Met^158^ allele is associated with a relatively smaller hippocampal and prefrontal volume compared with Val^158^ allele carriers (Honea *et al*., 2009). According to previous findings, the pre-existing smaller hippocampal volume might predispose individuals toward the development of maladaptive stress responses when exposed to a severe and sustained stressor (Vachon-Presseau *et al*., 2013). The association between CT experience and decreased FC in the APS haplotype may also indicate a vulnerability to stress that is related to the Met^158^ allele.

Like the LPS haplotypes, the HPS haplotype codes for the Val^158^ variant but have different mRNA secondary-structures that lead to almost a 20-fold reduction in enzymatic activity (Nackley *et al*., 2006). The limited metabolic capacity of the HPS haplotypes could result in a pronounced, prolonged elevation of the synaptic dopamine level. According to the inverted U-shaped modulation of the relationship between brain structures and functioning of the dopamine system, the excessive dopamine level might impair neuronal integrity and survival. Hence, the LPS haplotypes (LPS/LPS, LPS/APS) may be also vulnerable to childhood adversity, and the reduction in hippocampal FCs with CT experience, as found for APS haplotypes, may contribute to an inability to suppress behaviors with negative consequences, such as aggression.

We also found a conditional mediation effect of the hippocampal FC in the link between CT experience and the aggressive behaviors that were moderated by COMT. Consistent with previous studies, adverse experience and COMT have an interactive effect on impulsive aggression in early adolescents (Zhang *et al*., 2016). Siever (2008) posited that aggressive behavior might be a consequence of an imbalance between the top-down control or “brakes” provided by the OFC and excessive bottom-up “drives” triggered or signaled by the limbic regions. OFC damage or dysfunction could cause a lack of emotional control, which, in turn, could result in impulsive and aggressive behaviors (Gansler *et al*., 2009). Activation in the HIP is associated with the extinction of conditioned fear (Maheu *et al*., 2010). Considering the differences in the effects of CT experience in the various COMT haplotype individuals in our study and considering the involvement of the HIP in emotion regulation and adverse stimuli processing, we speculate that the CTQ × COMT interaction influences aggressive behavior through the HIP–OFC FC. Other shreds of evidence have also been found to support this suggestion. First, the two genotypes of COMT Val158Met have different effects on emotional reactivity to aversive stimuli (Montag *et al*., 2008). Moreover, the interaction between this polymorphism and early life stress contribute to susceptibility to neuropsychiatric disorders related to emotional dysregulation (Hosang *et al*., 2017). Second, the interaction effect between CT experience and COMT Val158Met influences fear conditioning and extinction (Deslauriers *et al*., 2018).

In our study, we found that only the right HIP–right OFC connectivity mediated the association between gene–environment interaction and aggressive behavior, a finding that was consistent with previous studies that showed a right laterality effect on the association between dopamine release and impulsivity in the limbic regions (Oswald *et al*., 2007) as well as a specific relationship between the right OFC and aggression in schizophrenia patients (Hoptman *et al*., 2002). Moreover, dopamine levels in the right hemisphere are higher than in the left side in rats (Afonso *et al*., 1993). However, the rightward OFC asymmetry in aggression is still up for debate, and one previous study reported a correlation between the left OFC gray matter volume and aggression ratings (Gansler *et al*., 2009). Hence, further studies are warranted to investigate the specific effect of the HIP–OFC coupling on aggressive behaviors.

The strengths of this study include that its design provided an opportunity to examine the mediating effects of hippocampal function on the relationship between gene, environment, and aggressive behavior. In addition, the haplotypes we considered are groups of alleles, which can show stronger linkage equilibrium and carry more information about the underlying functional variants; in turn, this could show a stronger association with behavior than individual variants and improve the statistical power in subsequent imaging genetic analyses (Qiu *et al*., 2015). A weakness of our study is that the childhood trauma questionnaire was a retrospective and self-report measurement, which might have led to inaccuracies caused by reporting bias or forgetfulness. However, a previous study suggests that retrospective self-reports tend to underestimate rather than overestimate actual situations (Hardt & Rutter, 2004). This might also be a possible reason for the undetected interactive effects on aggressive behavior.

In conclusion, these findings provide the first evidence that hippocampal function is the underlying mechanism for the relationship between the CTQ–COMT interaction and aggression. This study contributes to a better understanding of the influence of childhood experience on aggressive behavior in different genetic backgrounds.

## Data Sharing and Data Accessibility

The data that support the findings of this study are available from the corresponding author upon reasonable request.
